# Benign Breast Cyst in a Young Male

**DOI:** 10.7759/cureus.4814

**Published:** 2019-06-03

**Authors:** Nima Azimi, Azniv Azar, Ashraf Khan, Carolynn M DeBenedectis

**Affiliations:** 1 Department of Radiology, University of Massachusetts Medical School, Worcester, USA; 2 Department of Pathology, University of Massachusetts Medical Center, Worcester, USA

**Keywords:** radiology, breast, breast diseases, breast pathology, benign cysts, non-malignant breast disease

## Abstract

Simple benign breast cysts are commonly diagnosed in female breasts and may present as palpable masses. However, they are extremely uncommon in the male breast and are rarely reported in the literature. Here, we report a case of a simple benign cyst of the breast in a relatively healthy 37-year-old man. The patient initially presented with a palpable 2-3 mm tender left breast lump. Further evaluation with mammography and ultrasound revealed a mass most consistent with a simple benign cyst. However, considering the rarity of breast cysts in males, the lesion was biopsied to rule out malignancy. Pathology results from ultrasound-guided core needle biopsy demonstrated fibro-adipose tissue with a benign cyst lined by foamy cells with apocrine features, consistent with a diagnosis of a benign epithelial cyst and concordant with the radiological findings. To our knowledge, this is the youngest case of a benign breast cyst in a male that has been reported in the literature. In this case report, we discuss the typical features and presentation of breast cysts in males, associated imaging findings on mammography and ultrasound, and the necessity for pathological confirmation with biopsy in this population.

## Introduction

Breast cysts are fluid-filled sacs found within the breast. They are one of the most common types of breast masses, found mainly in females. These lesions are most common in pre-menopausal women in their 30s or 40s and typically disappear after menopause but may persist or reappear. Breast tissue is particularly sensitive to fluctuating levels of estrogen and progesterone during the menstrual cycle. Furthermore, fluid is produced and absorbed by the breast as part of this cycle. Given these hormonal differences in addition to histological differences, breast cysts in males are extremely rare. Considering the rarity of this entity in males, it is important to discuss the typical features and presentation of breast cysts in males and the associated imaging findings on mammography and ultrasound.

## Case presentation

The patient is a 37-year-old African American male with no significant past medical history, who presented with a painful left breast lump. He noted pain with palpation and with certain movements. The patient denied nipple discharge, fevers, chills, night sweats, fatigue, or weight loss and had no known family history of breast or ovarian cancer. On physical exam, there was a 2-3 mm palpable lump in the left breast at the 3 o’clock position with no nipple retraction. Examination of the right breast was unremarkable. Cervical, supraclavicular, and axillary lymph nodes were not palpable. A mammogram and ultrasound were ordered for further characterization of the lump.

Investigations/imaging findings

**Figure 1:**
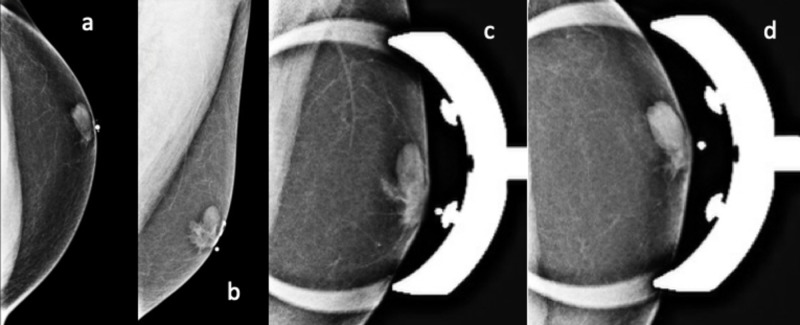
2D Digital Diagnostic Mammogram of Left Breast A 37-year-old man with a benign simple breast cyst: 2D digital diagnostic mammogram of left breast, CC (a), MLO (b), CC with spot compression (c), and MLO with spot compression (d) views. His diagnostic mammogram demonstrated an oval circumscribed mass within the left breast, just superior and lateral to the nipple. Bilateral gynecomastia is also seen with flame-shaped, subareolar fibroglandular densities. _2D - 2-Dimensional_ _CC - Cranial Caudal_ _MLO - Medial Lateral Oblique_

A bilateral 2D digital diagnostic mammogram revealed an oval circumscribed mass within the left breast, just superior and lateral to the nipple. Bilateral gynecomastia was also noted (Figure 1).

**Figure 2:**
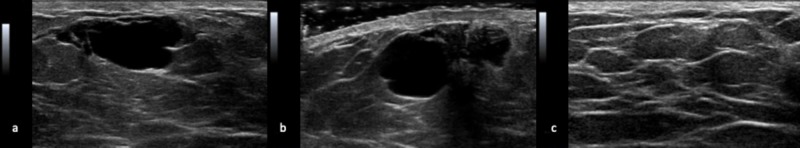
Left Breast Ultrasound of Benign Simple Cyst A 37-year-old man with a benign simple breast cyst: Left breast ultrasound (using 12 MHz linear transducer) images in the transverse (a) and longitudinal (b) axis demonstrates a simple anechoic cyst measuring 12 x 8 x 15 mm at the 12 o'clock position, corresponding to the palpable area with adjacent gynecomastia. The cyst was noted to disappear on ultrasound after biopsy of the cyst wall (c). _MHz - Mega Hertz_

A left breast ultrasound examination demonstrated a simple cyst measuring 12 x 8 x 15 mm at the 12 o'clock position, corresponding to the palpable area with adjacent gynecomastia (Figure 2).

These imaging findings were consistent with a simple benign breast cyst and categorized as Low Suspicion for Malignancy (Bi-RADS (Breast Imaging-Reporting and Data System) category 4A) because, given the rarity of cysts in male patients, a biopsy was recommended for confirmation.

The patient subsequently underwent an ultrasound-guided core needle biopsy. The cyst was noted to disappear on ultrasound after biopsy of the cyst wall, and a small amount of milky fluid was seen within the hub of the introducer. A post-procedure mammogram showed resolution of the mass, and only the coil clip and gynecomastia were seen.

Pathology

**Figure 3:**
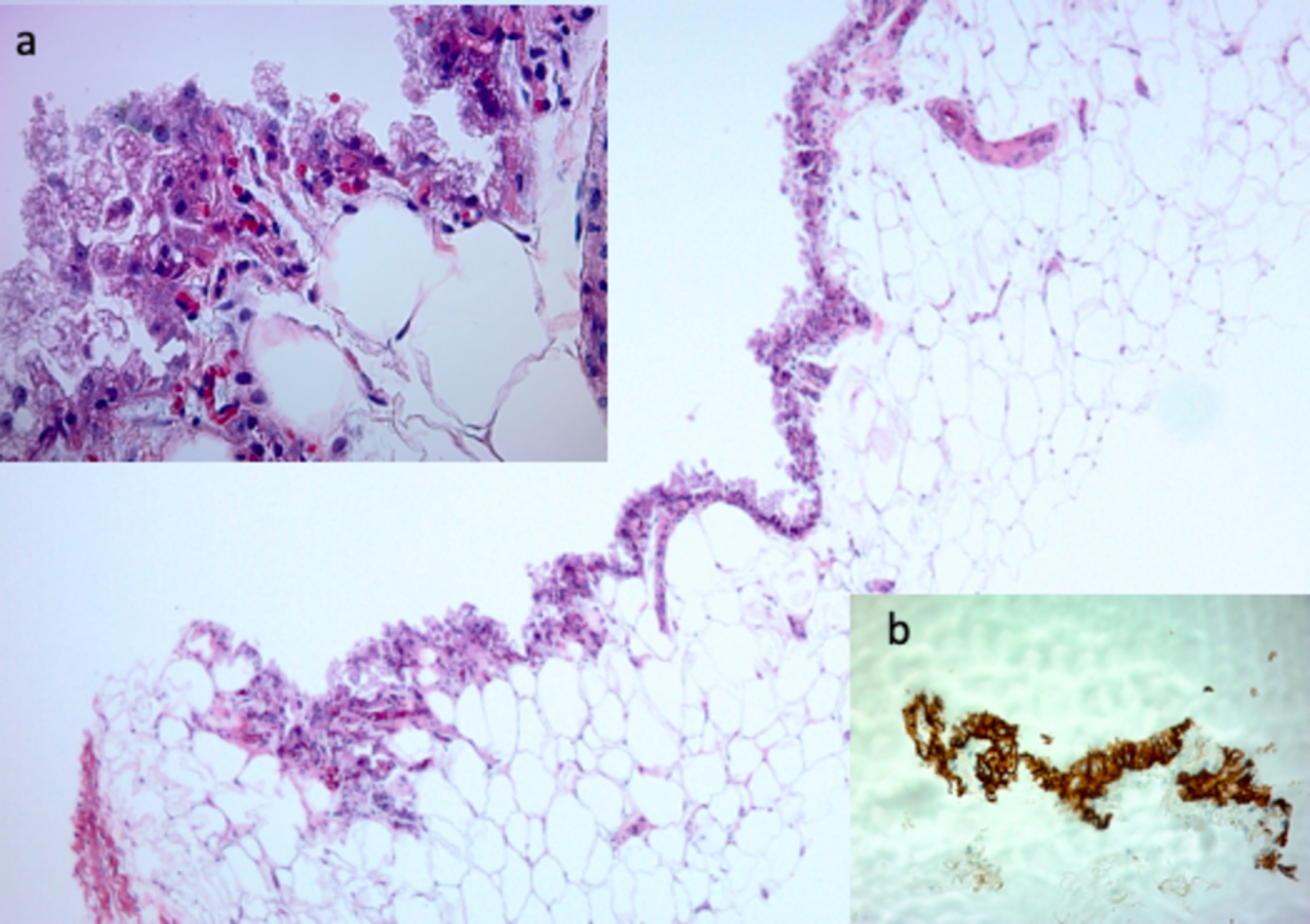
Left Breast Biopsy of a Benign Simple Cyst A 37-year-old man with a benign simple breast cyst: Photomicrographs of the tissue samples from a core needle biopsy show benign fibroadipose tissue and a simple cyst lined by a single layer of foamy cells that show apocrine and lactation-like features (4×, hematoxylin and eosin). Inset (a): These cells have uniform, round nuclei with prominent nucleoli and abundant clear, finely granular, and vacuolated cytoplasm (40x, hematoxylin and eosin). Inset (b): Immunohistochemical stains were performed and the lining cells are positive for Pan-cytokeratin (CK AE1/3) immunostain, which supports the epithelial nature of the cyst and negative for CD 68, a histiocytic marker (4x, cytokeratin staining). ^CK- Cytokeratin^ ^CD- Cluster of Differentiation^

Pathological examination of the core biopsy specimen demonstrated fibro-adipose tissue with a benign cyst lined by foamy cells with apocrine features and lactational changes, consistent with a diagnosis of benign epithelial cysts. Immunohistochemical stains demonstrated cells positive for Pan-cytokeratin (CK AE1/3) immunostain, which supports the epithelial nature of the cyst. In addition, these cells were negative for CD 68, a histiocytic marker. There was no evidence of malignancy or carcinoma (Figure 3).

Treatment

Considering the benign nature of the lesion as determined by pathological, radiological, and clinical correlation, no further follow-up was recommended.

Outcome and follow-up

At the time of publication, the patient is alive and well. He has not received any further therapy for his benign cyst.

## Discussion

Breast cysts are fluid-filled sacs found within the breast. They are one of the most common types of breast masses, found mainly in females. These lesions are most common in pre-menopausal women in their 30s or 40s and typically disappear after menopause but may persist or reappear. Breast tissue is particularly sensitive to estrogen and progesterone levels during the menstrual cycle. Additionally, fluid is produced and absorbed by the breast as part of this cycle. Estrogen replacement therapy is a known risk factor in women [1].

Breast cysts may present as tender palpable masses with discharge but are usually asymptomatic and found incidentally on imaging, typically, mammography or ultrasound [2]. Although most cystic lesions can be dismissed as benign simple cysts, complex cystic and solid masses usually require biopsy for a pathological examination to rule out malignancy [3].

On mammography, cysts may be seen as solitary or multiple, unilateral or bilateral, low or equal density masses with round, oval, or occasionally lobulated borders. Their margins are usually well-circumscribed but may be partly obscured by breast parenchyma [3]. On ultrasound, cysts are typically seen as well-circumscribed anechoic masses with posterior acoustic enhancement [4].

Mammography of the male breast accounts for less than 1% of mammographic examinations. It follows then that male breast cysts are extremely rare. This may be explained by the difference in histological features between the male and female breast in addition to hormonal differences, as explained above. Female breast tissue normally consists of ducts, stroma, and glandular tissue. In contrast, normal male breast tissue consists mainly of subcutaneous fat and a small number of ducts with minimal stroma. Given the lack of lobular structures in male breasts, the incidence of simple cysts and other lobular lesions are much lower in comparison [5].

The most common male breast mass is gynecomastia, followed by lipoma and epidermal inclusion cysts [6]. Lipoma was ruled out in this case due to the absence of fat density or a fatty mass on imaging. Additionally, since the lesion was not located in the dermis, an epidermal inclusion cyst was ruled out.

Our patient had imaging features that were most consistent with a benign simple breast cyst. In a female patient, there would not have been a need for a biopsy given the presence of the classic benign features of a simple cyst. However, considering the rarity of breast cysts in males, a biopsy was performed to rule out malignant disease.

To date, only two radiologic cases of benign breast cysts in males have been reported. Chantra et al. reported a case of a benign breast cyst in a 72-year-old male associated with a three-year history of intermittent bloody discharge and tenderness [7]. In addition, Parsian et al. reported the first case of a benign cyst in a 58-year-old male without gynecomastia [8]. Other studies reporting the radiologic features of male breast disease have not discussed benign simple breast cysts or reported any cases of such cysts [6,9-13]. In this case, our patient is a relatively healthy and young 37-year-old male with gynecomastia who presented with a tender breast lump and was subsequently found to have a benign simple cyst on mammography. To our knowledge, this is the youngest case of a benign breast cyst in a male that has been reported in the literature. Of note, this patient was also found to have bilateral gynecomastia on clinical examination. Robertson et al. have previously reported the theory that benign cysts in men could possibly have started as gynecomastia and are, therefore, a morphological subtype of this condition [9]. However, the most recent case of a benign breast cyst in a male had no associated gynecomastia; therefore, a definitive correlation cannot be drawn [8].

## Conclusions

This report highlights the rarity of simple cysts in males and their radiological features. Nevertheless, the management of these benign lesions requires pathological confirmation with biopsy to rule out malignancy, given their low incidence and prevalence in males.
